# Peer victimization and physical activity: the mediating role of self-esteem and physical self-concept

**DOI:** 10.3389/fpsyg.2026.1723909

**Published:** 2026-04-29

**Authors:** Yanlan Guo, Jian Wu, Jianmei Cui, Lei Yu, Yuze Zhang, Yinghai Liu, Syed Ghufran Hadier

**Affiliations:** 1 School of Physical Education, North University of China, Taiyuan, Shanxi, China; 2 School of Physical Education, Hebei Normal University, Shijiazhuang, Hebei, China; 3 College of Physical Education, Shanxi University, Taiyuan, Shanxi, China

**Keywords:** children and adolescents, peer victimization, physical activity, physical self-concept, self-esteem

## Abstract

**Introduction:**

Peer victimization (PV) is associated with physical activity (PA) in children and adolescents, potentially through psychological factors such as self-esteem and physical self-concept. However, the underlying mechanisms remain unclear. This study examined the association between PV and PA and the mediating roles of self-esteem and physical self-concept.

**Methods:**

A total of 1,063 students (mean age = 12.53 ± 1.75 years; grades 5–9) from twelve schools in Taiyuan completed the MPVS, PARS-3, SES, and Physical Self-Concept Scale. Associations and mediation effects were analyzed statistically.

**Results:**

PV was negatively associated with PA and with self-esteem and physical self-concept (*P* < 0.05). Self-esteem and physical self-concept were positively related to PA (*P* < 0.05). Both variables partially mediated the PV–PA relationship, accounting for 51.20% of the total effect.

**Discussion:**

PV negatively influences PA both directly and indirectly through self-esteem and physical self-concept. Interventions targeting victimization and improving self-related perceptions may help promote physical activity in youth.

## 1 Introduction

The multidimensional concept of Peer Victimization (PV) was first proposed by Mynard and Joseph (2000), which refers to the negative impact and harm of peer attacks on the mental health and development of attacked children and adolescents in various aspects (Zhang et al., 2009). It’s a negative peer relationship that happens in children and adolescents. According to data released by UNESCO in 2019, the global incidence of peer victimization is around 32% (Attawell, 2019). Foreign studies have shown that 5–15% of children and adolescents have suffered serious or persistent partner abuse (Graham et al., 2006; Zhao et al., 2016). Domestic research has found that 16.5% of children and adolescents suffer peer victimization, which is most common in primary and junior high school (Weimer and Moreira, 2014; Pérez Cortés, 2016). Peer victimization is a cause of mental illness (Moore et al., 2017), and adolescents subjected to peer victimization are prone to a series of internalization and externalization problems, which are manifested in poor academic performance, sleep difficulties, loneliness, anxiety, depression and even suicidal ideation, while externalization problems are manifested in aggression, social withdrawal and poor executive function (Craig and Pepler, 2007; Holt et al., 2015). Preference for sedentary items (such as screen time) is strongly associated with physical activity participation (Salvy et al., 2008; Salvy et al., 2009). The physical activity behavior of children and adolescents is related to peer relationship, such as peer acceptance, peer rejection and peer victimization. Peer rejection is a negative type of peer acceptance, and the level of peer rejection may be associated with peer victimization and may be the trigger for children and adolescents to suffer peer victimization (Chen, 2009). Children and adolescents with high levels of peer rejection have a higher risk of adaptive problems, such as aggression, social withdrawal, and learning avoidance. Peer victimization is a kind of negative peer experience. Studies have found that peer victimization has a negative impact and harm on the physical and mental development and social adjustment of children and adolescents, resulting in long-term maladjustment, such as withdrawn personality, emotional disorders, social withdrawal, poor executive function, etc., resulting in behavioral disorders in sports activities as one of the externalizing problems (Stankov et al., 2012). Relevant studies have shown that the incidence of peer victimization is related to the participation rate of physical activity, especially among overweight and obese children and adolescents (Henriksen et al., 2016; Roman and Taylor, 2013; Storch et al., 2007). Self-esteem and body self-concept are important determining factors for children and adolescents to engage in sports activities (Jekauc et al., 2017). Adolescents with higher self-esteem have stronger motivation to participate in sports activities (Altintaş and Aşçi, 2008), and body self-concept is closely related to the behavior of children and adolescents. Reduced physical activities tend to be associated with self-esteem and physical self-concept, which are important determining factors for children and adolescents to engage in physical activities (Jekauc et al., 2017). Adolescents with higher self-esteem have stronger motivation to participate in physical activities (Altintaş and Aşçi, 2008), and physical self-concept is closely related to the behavior of children and adolescents. The reduction of physical activity tends to reduce the self-esteem and physical self-concept of children and adolescents, and children and adolescents who suffer peer victimization are characterized by low self-esteem and physical self-concept (Hawker and Boulton, 2000), which verifies that self-esteem and physical self-concept are one of the important protective factors of peer victimization (Boulton and Smith, 1994; Grills and Ollendick, 2002; Tambelli et al., 2012; Diamantopoulou et al., 2008). Self-esteem and physical self-concept play an important role in the growth of children and adolescents, and self-esteem is a psychological trait at a higher level than physical self-concept. Longitudinal studies show that there is a causal relationship between self-esteem and self-concept, and self-esteem may be related to individual self-concept (Tan, 2020). In a word, peer victimization may be associated with a decrease in physical activity of children and adolescents (Stearns et al., 2017). Physical activity is related to the self-esteem and physical self-concept of children and adolescents, and individuals with low self-esteem and physical self-concept are prone to peer victimization.

## 2 Literature review

### 2.1 The influence of peer victimization on physical activity

The experience of peer victimization in adolescents has a serious impact on the later development process, and such painful experiences can leave lasting scars in adulthood (McDougall and Vaillancourt, 2015). Children and adolescents suffer peer victimization at the golden age of 8–15 years of growth and development, and adult male individuals will show a greater tendency of aggression and a higher probability of crime, while adult women will show more internalization problems (Renae McGee et al., 2011; Gibb et al., 2011).Stearns divided 18, 157 middle school students in 43 Canadian high schools by BMI level, and then analyzed peer victimization, screen time and moderate intensity physical activity through multi-level pathway analysis. The results found that peer victimization is related to the motivation of middle school students at any BMI level to participate in physical activity. In addition, screen time increased, which was more significant in the overweight BMI group and the obese group, and the survival of men and women was significantly different (Stearns et al., 2017). Vanderwater found that overweight or obese adolescents are prone to peer victimization, spend less time on friends, spend less time with friends, and socialize less, resulting in less activity, less activity, and longer screen time such as watching TV (Vandewater et al., 2015). Similar studies have found that boys and girls who experience bullying are less motivated to engage in physical activity and are more likely to choose sedentary behaviors (Liu et al., 2024). Smith et al found that peer support is positively correlated with children’s motivation and level of physical activity (Smith, 1999; Bungum and Vincent, 1997). However, few studies have explored the relationship between external negative factors (such as peer victimization, parental distress, depressive symptoms) and physical activity disorders (Gray et al., 2008). Muchicko conducted a questionnaire survey on peer victimization, physical activity behavior and attitude toward physical activity among 80 adults, and found that peer victimization has a negative relationship with physical activity behavior and attitude (Muchicko, 2012). Storch et al found that in the sample of overweight children, a higher level of peer victimization could be associated with a lower level of physical activity (Storch et al., 2007).

### 2.2 The mediating role of self-esteem

Self-esteem has a profound impact on all aspects of an individual (Branden, 1995), among which it plays an extremely important role in the growth of children and adolescents and constitutes the basis of self-belonging (Rosenberg, 1979). Self-esteem refers to how a person views and evaluates himself, reflecting how much he values himself. This evaluation is based on the performance of adolescents in work, study and life (Donnellan et al., 2011). Relevant studies have shown that self-esteem can be related to mental health and a series of important life areas, such as anxiety, depression, social relationship satisfaction and health (Orth et al., 2012; Sowislo and Orth, 2013). High self-esteem is the core psychological source of all kinds of positive behaviors, and low self-esteem is the root of personal problems, which leads to social problems and dysfunction, and has obvious implications for intervention at the individual and social levels. Among them, peer victimization of children and adolescents has a particularly prominent impact on self-esteem.

Adolescents’ peer relationship is regarded as a predictor of current and future psychosocial development (Egan and Perry, 1998), and many studies on peer victimization focus on its impact on self-esteem of children and adolescents (Hawker and Boulton, 2000). Academic circles agree that adolescents who have experienced peer victimization have lower self-esteem than those who have not. Yang conducted a questionnaire survey on peer victimization, peer acceptance and self-esteem scale among 392 middle school students, and the data analysis showed that peer acceptance and self-esteem of middle school students who have experienced peer victimization have declined, regardless of gender. And are more likely to be rejected by their peers (Yang and Doh, 1999). Tsaosis conducted a meta-analysis of 121 relevant studies on peer victimization, bullying behavior and self-esteem, and found that peer victimization was negatively correlated with self-esteem (*r* = −0.47), and bullying behavior was also negatively correlated with self-esteem (*r* = −0.07), but the correlation coefficient was small (Tsaousis, 2016). Overbeek et al. (2010) examined the two-way and longitudinal relationship between peer victimization and self-esteem in adolescents, and examined the moderating role of personality types of insufficient control, over-control and self-resilience in relationships. A longitudinal study was conducted on 774 adolescents aged 11–16 years, with data collected three times every one year. The structural equation model analysis of Mplus showed that higher peer victimization was associated with lower self-esteem among under-controlled and over-controlled adolescents. However, low self-esteem did not predict subsequent self-reported victimization (Overbeek et al., 2010).

The level of self-esteem of children and adolescents may be associated with the enthusiasm of participating in sports activities (Biddle and Asare, 2011; Zurita-Ortega et al., 2018). Richman et al used the SES questionnaire to investigate the self-esteem levels of 60 karate students. The results show that when the karate level is the same, the students who score higher on the self-esteem scale participate in this sport for a longer time (Richman and Rehberg, 1986). Altintas analyzed the differences between multi-dimensional self-esteem and sports activity participation of 803 adolescents in Turkey, and found that adolescents with relatively high self-esteem had stronger motivation to participate in sports activities (Altintaş and Aşçi, 2008). Lindwall longitudinally investigated the relationship between overall self-esteem, body self-recognition and physical activity of 705 Canadian girls, followed them for two years and measured them three times, and found that physical self-recognition, self-esteem and physical activity changes were positively correlated (Lindwall et al., 2014). Therefore, peer victimization is proposed to be associated with physical activity level through self-esteem as research hypothesis 1.

### 2.3 The mediating role of body self-concept

According to Myers’ definition, self-concept is the question “Who am I?” the set of answers to this question (Myers, 2015). There are many dimensions of self-concept, which interact with individual perception ability, physical health, physical function and mental health (Palomino-Devia et al., 2018). Shavelson’s multi-dimensional and multi-level theoretical model of self-concept and Fitts’s Tennessee multi-dimensional theoretical model of self-concept are more influential in the academic circle. Since peer victimization is related to adolescents’ physical activity behaviors and is an influence of externalization, this study referred to the externalization reference index in Shavelson’s multidimensional self-concept model, and selected physical self-concept as the main representative indicator of self-concept for discussion (Shavelson et al., 1976). Body self-concept refers to a person’s perception of their own appearance and physical abilities, and at its core is the ability to accept contradiction and reconcile with themselves. Low physical self-concept, will produce a sense of inferiority, uncertainty and doubt about themselves lead to lack of courage, cannot fully develop their own potential, thus missing opportunities. People with a strong physical self-concept think that they are smart, excellent, more independent, secure and accomplished, and have a high degree of self-identification, which makes them more likely to achieve success than people with a weak sense of self-identification (Hu et al., 2022). The core task of adolescent growth is to improve self-concept, and exercise is a new space for them to seek self-identification and self-perception (Wang et al., 2021).

There is no doubt about the influence of peer victimization on children and adolescents’ self-concept. Many studies have shown that self-concept can be related to a person’s behavior pattern, especially play a mediating role in important behavior results, in which self-concept mediates the relationship between peer victimization and academic performance, and also mediates the relationship between motor skills and mental health (Chen et al., 2015; Viholainen et al., 2014; Lohbeck and Petermann, 2017). Self-concept and loneliness play a moderating and mediating role between personality traits and school bullying (Zhang et al., 2021). Self-concept is closely related to problem behaviors of children and adolescents, and low self-concept is one of the factors for adolescents to be bullied and bully others (Diamantopoulou et al., 2008). Relevant research shows that children who experience peer victimization usually show: lack of security, cannot make a correct evaluation of self, feel inferior, depression, antagonism and rebellious psychology. Most of these psychological problems are caused by a lack of self-concept. In particular, the teenagers who suffer peer victimization are in a period when the trust system is seriously damaged and the normal social interaction is lacking. It will have a negative impact on their social ability and personality (McCarthy et al., 2018). Norrington et al verified the effect of peer victimization on mental health through self-concept through a questionnaire survey of 1,413 adolescents (Norrington, 2021). Jenkins and Lohbeck’s study showed that students who had experienced peer victimization had worse academic performance than those who had not, and self-concept mediated the relationship between peer victimization and academic performance (Lohbeck and Petermann, 2017; Zhang et al., 2021; McCarthy et al., 2018; Norrington, 2021; Jenkins and Demaray, 2012). Viholainen et al selected 327 adolescent girls aged 12–16 to verify whether body self-concept mediates motor skills and mental health, and verified this mediating effect through structural equation model (Viholainen et al., 2014).

Benitez-Sillero et al measured the physical activity, bullying questionnaire and physical self-concept of 870 adolescents aged between 12 and 19, and found that adolescents’ physical self-concept mediated the relationship between bullying and physical activity participation (Benítez-Sillero et al., 2022). It suggests that body self-concept in adolescence can be related to physical activity and sports participation. According to Hart’s competency motivation theory, competency motivation increases when a person successfully masters a task (Harter, 1987). Successful mastery of the task promotes the perception of self-competence as well as the concept of self, thus encouraging the person to engage in further activities. This theory has been applied in the context of youth participation in sports, proving that body self-concept is an important motivational factor to participate in and maintain physical activity (Jackson and Marsh, 1986). Jekauc et al verified that young people’s sports ability is related to their participation in sports activities through physical self-concept, which is an important determinant of adolescents’ physical activities (Jekauc et al., 2017). Onetti measured physical activity and self-concept among 40 adolescents aged 16–20 from Spain, and found that physical self-concept was positively correlated with physical activity level and negatively correlated with sedentary behavior of adolescents (Onetti-Onetti et al., 2019). Therefore, it is proposed that body self-concept mediates peer victimization and physical activity level as research hypothesis 2.

**FIGURE 1:**
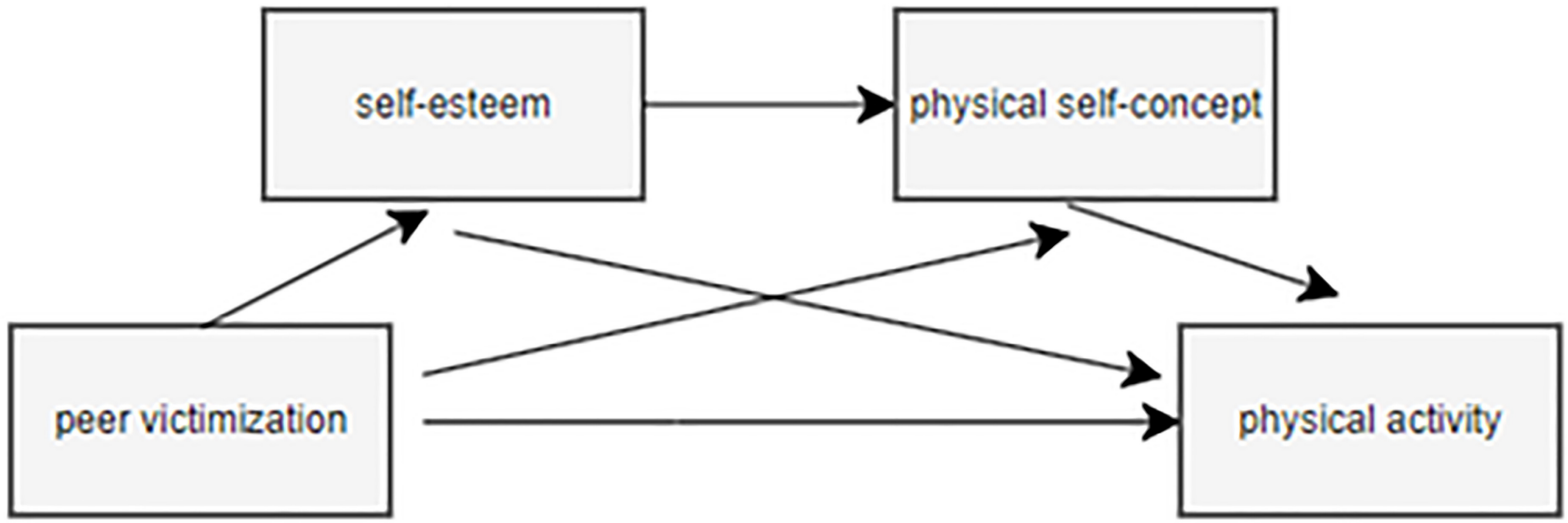
The hypothetical chain mediation model between peer victimization and physical activity.

Self-esteem can be positively associated with body self-concept and is indirectly related to body self-concept through self-acceptance. Physical activity level is also related to both self-esteem and body self-concept. Previous studies have confirmed that peer victimization is related to physical activity among children and adolescents (Storch et al., 2007; Liu et al., 2024), and that this relationship may operate through self-esteem or body self-concept (Zurita-Ortega et al., 2018; Palomino-Devia et al., 2018; Jackson and Marsh, 1986). However, most existing studies have examined these variables independently or considered only a single mediator, and the potential sequential mechanism linking self-esteem and body self-concept remains underexplored. From a theoretical perspective, self-esteem reflects a global evaluation of the self, whereas body self-concept represents a domain-specific perception related to physical attributes. According to the hierarchical structure of the self-system, global self-evaluations can influence more specific domains of self-perception. Therefore, peer victimization may first affect individuals’ overall self-esteem, which subsequently shapes their body self-concept, ultimately influencing their engagement in physical activity. Based on this reasoning, this study proposes a chain mediation model in which self-esteem and body self-concept operate as sequential mediators between peer victimization and physical activity. This approach extends previous research by integrating multiple psychological mechanisms into a unified analytical framework and provides a more comprehensive understanding of how negative peer experiences influence adolescents’ behavioral outcomes. Therefore, hypothesis 3 is proposed: self-esteem and body self-concept function as serial mediators in the relationship between peer victimization and physical activity (see Figure 1).

## 3 Materials and methods

### 3.1 Sample size

By random sampling, 1,200 children and adolescents from grades 5 to 9 from 12 primary and middle schools (6 primary schools and 6 middle schools) in 6 districts of Taiyuan were selected as the investigation objects, of which 623 were boys (51.92%) and 577 were girls (48.08%). The mean age was 12.53 ± 1.75 years. A total of 137 invalid questionnaires were excluded and 1063 valid questionnaires were retained. The effective rate of the questionnaire was 88.58%, among which 136 subjects suffered peer victimization.

### 3.2 Measuring tool

#### 3.2.1 Measurement of peer victimization

The physical aggression and relational aggression subscales modified by Zhang Wenxin et al. on the multidimensional peer victimization scale MPVS compiled by Mynard and Joseph were used to measure peer victimization of children and adolescents, with a total of 11 items (Mynard and Joseph, 2000). The Chinese version of questionnaire is a more widely used measurement tool in the current research on peer victimization of children and adolescents. Zhang Wenxin’s relevant research shows that this scale is suitable for measuring peer victimization of children and adolescents in the Chinese cultural background (Zhang et al., 2009), with a 4-point scale ranging from 0 to 3. The higher the score, the more serious the peer victimization, the better the half-score and retest reliability of this scale. The reliability of the physical aggression scale and the relational aggression scale Cronbach’s Alpha were 0.792 and 0.816, respectively.

#### 3.2.2 Physical activity measurement

The physical activity Rating Scale (PARS-3) revised by Liang Deqing et al., was used to evaluate the participants’ physical exercise in the previous month from the three aspects of exercise intensity, time and frequency (amount of exercise = intensity * time * frequency). Each aspect is divided into 5 grades, intensity and frequency from 1 to 5 grades are 1–5 points, time from 1 to 5 grades are 0–4 points, so the highest score is 100 points, the lowest is 0 points. Small amount of exercise: ≤ 19 min; Moderate exercise: 20–42 min; Large exercise: ≥ 43 points. The retest reliability of this scale was good, Cronbach’s Alpha was 0.828.

#### 3.2.3 Self-esteem measurement

Rosenberg Self-esteem Scale (SES) Chinese version, used to assess the level of self-worth and self-acceptance of individuals, a total of 10 items, the scale is scored in a 4-point scale, from “strongly disagree” to strongly agree, 1–4 points. A higher score indicates a higher level of self-esteem. Cronbach’s Alpha in this study was 0.793.

#### 3.2.4 Body self-concept measurement

The adolescent body self-scale compiled by Huang Xiting and Chen Hong in 2002 was used, with a total of 33 items, of which 11 items were physical features, 9 items were athletic features, 6 items were physical features, 4 items were sexual features and 3 items were negative features. The scale was scored with 7 points, with 1–7 points indicating “very dissatisfied,” “dissatisfied,” “relatively dissatisfied,” “general,” “relatively satisfied,” “satisfied” and “very satisfied,” respectively. The higher the total score, the more satisfied the subjects were with their bodies, and the higher the body self-concept. The selection of localization scale is more suitable for our cultural background. According to the characteristics of junior middle school students and the purpose of this study, and interviews with mental health teachers and experts, it is suggested to delete the 4 items of sexual characteristics, so a total of 29 items are actually issued in the questionnaire. GFI = 0.917, CFI = 0.943, and TLI = 0.922 were all greater than 0.9 and close to 1; AGFI = 0.851 was greater than 0.85; and RMSEA = 0.073 < 0.08, Cronbach’s αfor all four dimensions was greater than 0.7, CR was greater than 0.7, AVE was greater than 0.5, item-total correlations were greater than 0.3, and inter-item correlations were greater than 0.3, the Cronbach’s α reliability coefficient for this questionnaire is 0.853.

### 3.3 Statistical analysis

SPSS25.0 was used to conduct preliminary data sorting and analysis and *t*-test. Then AMOS23.0 was used to analyze the correlation among the variables of peer victimization, physical activity, self-esteem and body self-concept. The mediating effect was tested by deviation-corrected non-parametric percentile Bootstrap method. Finally, a chain model of multiple mediation effects is constructed. All path coefficients reported in the structural equation model are standardized estimates (β).

## 4 Results

All reported path coefficients are standardized coefficients (β). Table 1 describes the mean and standard deviation of all study variables, as well as gender differences.

**TABLE 1:** Descriptive statistics of all variables.

variables	Male		Female		Subject		*P*
	Mean	SD	Mean	SD	Mean	SD	
Peer victimization	1.63	1.42	1.89	1.55	1.76	1.49	0.177
Self-esteem	18.29	2.42	21.83	2.51	20.06	2.47	0.031*
Physical self-concept	69.88	9.75	73.17	9.32	71.53	9.54	0.000***
Physical activity	18.65	3.35	16.21	3.23	17.43	3.29	0.026*

*P < 0.05; ***P < 0.001.

### 4.1 Correlation analysis of self-esteem, body self-concept, physical activity and peer victimization

Table 2 shows the Spearman Rank correlation results among peer victimization, self-esteem, body self-concept, and physical activity variables. There was a significant negative correlation between peer victimization and physical activity (*r* = −0.53; *p* < 0.05); Physical activity was positively correlated with self-esteem and body self-concept (*r* = 0.49, *r* = 0.34; *p* < 0.05); There was a significant positive correlation between self-esteem and body self-concept (*r* = 0.61; *p* < 0.01), peer victimization was negatively correlated with self-esteem and body self-concept (*r* = −0.29, *r* = −0.16; *P* < 0.01).

**TABLE 2:** Spearman Rank correlations for all variables.

variables	Peer victimization	Self-esteem	Physical self-concept	Physical activity
Peer victimization	1	1	1	1
Self-esteem	−0.29**			
Physical self-concept	−0.16**	0.61**		
Physical activity	−0.53*	0.49*	0.34**	

*P < 0.05; **P < 0.01.

### 4.2 The relationship between peer victimization and physical activity: a chain-mediated model test

**FIGURE 2:**
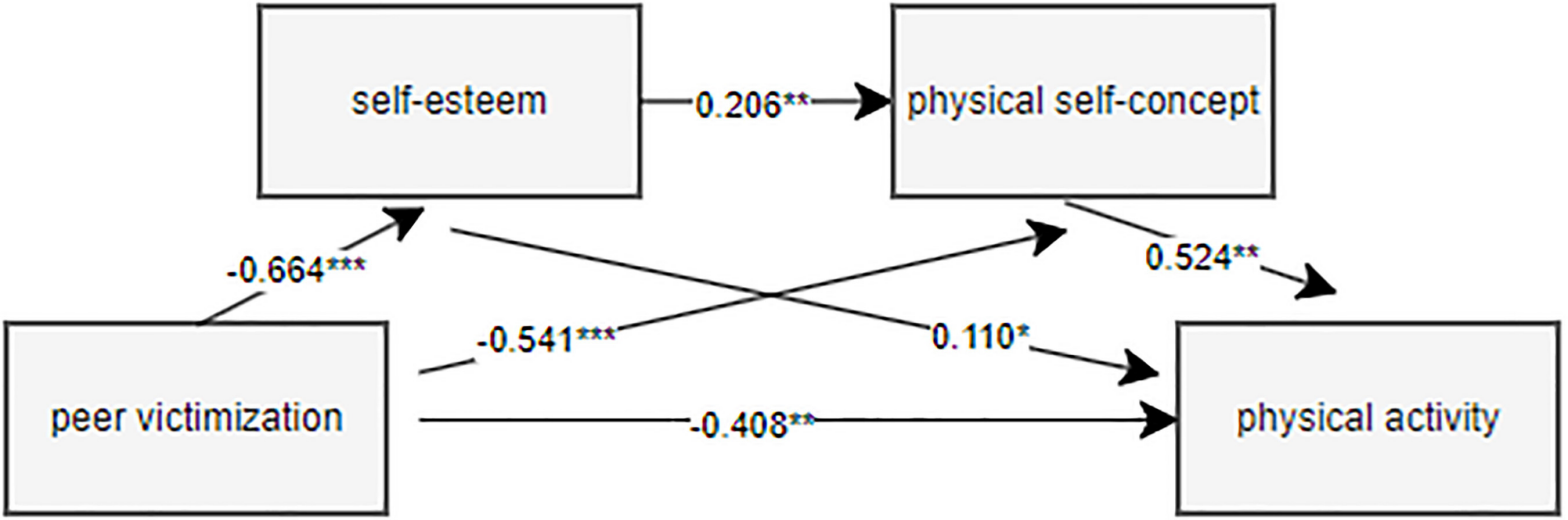
The final chain mediation model between peer victimization and physical activity.

Parameter estimation of structural equation model and mediation effect test were performed by variance maximum likelihood method and Bootstrap test (Liu et al., 2015; Wen et al., 2022). Peer victimization as a predictor, physical activity as an outcome variable, and self-esteem and body self-concept as mediating variables were used for path analysis. The fitting index of model M0 was obtained considering all the individual path relationships (Table 3). The results showed that the model was well fitted, and it was found that peer victimization → self-esteem → physical activity, peer victimization → physical self-concept → physical activity, peer victimization → self-esteem → physical self-concept → physical activity, peer victimization → self-esteem → physical self-concept → physical activity. Therefore, the revised model M1 is a chain-type multiple mediation model, as shown in Figure 2, which verifies the theoretical model in Figure 1.

**TABLE 3:** Fit indices of the hypothetical model and the competing model.

Model	χ^2^/df	CFI	SRMR	RMSEA
M_0_	3.30	0.96	0.03	0.05
M_1_	3.28	0.96	0.03	0.05

M0 is a hypothetical model, M1 is a competitive model.

According to Table 4, the total effect value of peer victimization on physical activity was −0.836; the indirect effect of peer victimization on physical activity was −0.408. The path coefficient of peer victimization on self-esteem was −0.664. The path coefficient of peer victimization on body self-concept was −0.541. The path coefficient of self-esteem on physical activity was 0.110; the path coefficient of physical self-concept to physical activity was 0.524. The path coefficient of self-esteem on body self-concept was 0.206. It can be seen from Table 5 that the 95% confidence interval of all the Bootstrap path coefficients does not include 0, indicating that H1, H2 and H3 are all valid. The indirect effects of the three mediation paths were significant, with effect sizes of 0.073, 0.283, and 0.072, accounting for 8.73, 33.85, and 8.62% of the total effect, respectively. The proportion of mediation effect represents the ratio of the indirect effect to the total effect.

**TABLE 4:** Regression analysis of a chain multiple mediation model between peer victimization and physical activity.

Predictor	Self-esteem			Physical self-concept			Physical activity			total effect		
	β	*SE*	*t*	β	*SE*	*t*	β	*SE*	*t*	β	*SE*	*t*
Peer victimization	−0.664	0.149	−4.451***	−0.541	0.06	−8.998***	−0.408	0.039	−10.391***	−0.836	0.054	−15.60***
Self-esteem				0.206	0.07	2.931**	0.110	0.045	2.479*			
Physical self-concept							0.524	0.044	11.705***			
R^2^	0.398			0.590			0.353			0.645		
R^2^ after adjustment	0.394			0.584			0.338			0.642		
F	88.58***			95.69***			24.01***			243.46***		

*p < 0.05, ** *p* < 0.01, *** *p* < 0.001.

**TABLE 5:** Analysis of the results of chain mediated effects.

Type	Effect	Boot SE	BootLLCI	BootULCI	Effect ratio
Total Effect C	−0.836	0.047	0.556	0.848	100%
Direct effect C’	−0.408	0.019	0.379	0.603	48.80%
Indirect effect 1: Peer victimization → self-esteem → physical activity	−0.073	0.007	0.051	0.077	8.73%
Indirect effect 2: Peer victimization → Physical self-concept → Physical activity	−0.283	0.025	0.332	0.431	33.85%
Indirect Effect 3: Peer victimization → Self Esteem → Physical Self Concept → Physical Activity	−0.072	0.005	0.003	0.021	8.62%
Total indirect effects	−0.428	0.023	0.364	0.529	51.20%

BootLLCI is the lower limit of the 95% interval for Bootstrap sampling, and BootULCI is the upper limit of the 95% interval for Bootstrap sampling.

The results show that peer victimization is negatively related to the physical activity of children and adolescents, that is, the higher the level of peer victimization, the lower the physical activity; peer victimization can be related to the physical activity of children and adolescents through self-esteem, physical self-concept or self-esteem and physical self-concept, that is, the higher the level of peer victimization of children and adolescents, the lower the level of self-esteem and physical self-concept, resulting in the lower level of physical activity.

## 5 Discussion

Through a survey of 1,063 primary and secondary school students, this study found that peer victimization is related to children’s and adolescents’ physical activity, and self-esteem and body self-concept play multiple mediating roles in the chain. Peer victimization was negatively correlated with physical activity, self-esteem and physical self-concept of children and adolescents. Peer victimization is associated with physical activity of children and adolescents, and self-esteem and physical self-concept of children and adolescents play an intermediary role in the relationship between peer victimization and physical activity. The research results confirm hypothesis 1, 2 and 3, which is consistent with previous studies (Lindwall et al., 2014; Benítez-Sillero et al., 2022). Previous studies mostly discussed the effects of self-esteem and body self-concept on peer victimization on physical activity as separate mediating variables. Based on the research on existing mediating variables, this study incorporated self-esteem and body self-concept as serial variables into structural equation model analysis, and explored a new path for the mechanism relationship between peer victimization and physical activity in children and adolescents.

Among children and adolescents, the conclusion that peer victimization is associated with to lower self-esteem in children and adolescents is consistent with the conclusion that self-esteem (Hawker and Boulton, 2000; Yang and Doh, 1999), body self-concept and physical activity are significantly positively correlated (Viholainen et al., 2014). If measures can be taken to improve the self-esteem and physical self-concept of the victims, the negative impact of peer victimization on physical activities can be weakened to some extent.

### 5.1 The relationship between peer victimization and physical activity

Peer victimization can be related to the physical activity of children and adolescents, and as shown by the path coefficient in Figure 2, the prediction ability is strong. Children and adolescents subjected to peer victimization have poorer executive function and social withdrawal, which may be closely related to participation in sports activities (Salvy et al., 2008; Salvy et al., 2008). Peer victimization at an early age may create an environment of insecurity that can lead to a decreased level of self-concept in adolescents, resulting in self-doubting thoughts such as “am I not good enough,” which may be a reason for adolescents to reduce physical activity. Peer support can promote the social behavior of children and adolescents, and physical activity is a kind of social action. In order to help children and adolescents who have suffered peer victimization, it is suggested that schools and families actively help to find peer support, remove barriers to physical activity, and improve their self-esteem and physical self-concept, so as to enhance the enthusiasm of children and adolescents in physical activity. Thereby reducing peer victimization (Faith et al., 2002).

### 5.2 The multi-mediating role of self-esteem and body self-concept in the chain between peer victimization and physical activity

This study found that in addition to the direct effect of peer victimization on physical activity, peer victimization is also related to physical activity through the self-esteem, body self-concept or self-esteem and body self-concept of children and adolescents, that is, self-esteem and body self-concept play a sequential mediating role between peer victimization and physical activity. This further confirms the results of previous studies on the relationship between self-esteem and body self-concept, indicating that self-esteem and body self-concept can promote children and adolescents to participate in sports activities.

Self-esteem can be related to some indicators of physical activity participation to a certain extent. The self-esteem level of male students is higher than that of female students, and the scores of male students’ physical activity frequency and multiple indicators are higher than those of female students (Chen, 2012). Physical self-esteem and perceived body value of female college students were significantly positively correlated with physical activity (*r* = 0.10, *r* = 0.15, *p* < 0.05). Self-esteem had little effect on physical activity level, and body self-esteem as an intermediary variable had little effect on physical activity as a whole. It is concluded that self-esteem can be related to college students’ physical activity through physical self-esteem, but it is not the main influencing factor (Xu, 2003), which is similar to the results of this study. In this study, the independent mediating effect of self-esteem is not significant, which is specifically reflected in the latter half of the mediating effect, and the predictive effect of self-esteem on physical activity is not significant. More studies have shown that participation in physical activity can increase self-esteem levels. It is recommended that people with low self-esteem participate in physical activity regularly and should not sit too much (Zamani Sani et al., 2016).

The indirect mediating effect of peer victimization on physical activity through physical self-concept is significant, and both the front and back segments of the mediating effect are significant, indicating that peer victimization reduces physical activity by reducing the level of physical self-concept of children and adolescents. Physical self-concept during adolescence can be associated with physical activity and sports participation. According to Hart’s competency motivation theory, competency motivation increases when a person successfully masters a task (Harter, 1987). Body self-concept is an important determinant of adolescents’ physical activities (Jekauc et al., 2017). Jenkins selected students from grades 3 to 5 as survey objects and found that self-concept and peer victimization were significantly negatively correlated (Jenkins and Demaray, 2012). The academic community also conducted studies on self-concept as an intermediary variable, for example, self-concept mediates the relationship between peer victimization and social support (Lohbeck and Petermann, 2017). Adolescents’ athletic ability is related to participation in physical activities through body self-concept, which confirms the results of this study (Jekauc et al., 2017).

Most previous studies only examined the single mediating effect of self-esteem or body self-concept on peer victimization and physical activity. This study explored the chain mediating effect of self-esteem and body self-concept, and confirmed that peer victimization is related to physical activity through self-esteem and body self-concept of children in adolescence. Both self-esteem and self-concept can alleviate individuals’ negative emotions. Studies have shown that children and adolescents with high self-esteem have a clearer self-concept structure, which is consistent with the results of this study (Overbeek et al., 2010; Benítez-Sillero et al., 2022). Longitudinal studies show that the causal relationship between self-esteem and self-concept is verified in both directions, but the cause-effect relationship between self-esteem and self-concept is stronger, and self-esteem is more likely to be related to individual self-concept, which confirms the sequential relationship between self-esteem and body self-concept (Chen, 2009). Similar mediation models have also been shown in other studies, self-esteem is the mediating variable of depression that is related to adolescent bullying, and the core task of adolescent growth is to improve self-concept.

### 5.3 The contribution, limitation and future research prospect of this study

This study explores the mechanism of the relationship between peer victimization and physical activity, mainly discusses the mediating effect of self-esteem and body self-concept in the relationship between the two, and takes self-esteem, a multidimensional concept, as a whole variable to explore the relationship between peer victimization and physical activity. Previous studies have linked the overall concept of self-esteem with sub-concepts such as body self-esteem to explore the relationship between peer victimization and physical activity. However, this study takes self-esteem and body self-concept as serial variables to explore the relationship between peer victimization and physical activity, opening up a new path. Peer victimization can be related to the physical activity of children and adolescents through self-esteem and physical self-concept. It has enriched the relevant research content of peer victimization and physical activity, further enriched the theoretical basis of peer victimization and physical activity, and also provided effective new ideas for preventing or intervening children and adolescents from peer victimization.

Limitations and future prospects of this study. First, the survey objects are mainly middle school students. Middle school is the key stage of students’ physiological and psychological development, and there is a great difference between male and female students. It is better to include gender as a moderating variable in the analysis. Second, peer victimization is explored to be associated with physical activity through self-esteem and body self-concept, and the results show that self-esteem has a significant mediating effect between peer victimization and physical activity, but the proportion of the effect is not large. The reason for this result may be that although self-esteem is regarded as a whole concept, the specific situation of multiple dimensions of self-esteem is not fully taken into account in the measurement. For example, physical fitness, athletic ability, physical condition and physical attractiveness should be taken into account when examining physical self-esteem. Thirdly, compared with longitudinal studies, cross-sectional designs have limitations in establishing causal relationships. Longitudinal study considers more factors, time interval and path coefficient are calculated more accurately, and the stability of mediating variables and regulating variables is higher. In the future, longitudinal research methods can be used to further explain the long-term changes in the relationship between peer victimization and physical activity.

## 6 Conclusion

Peer victimization is an important factor that is related to children and adolescents’ physical activity, and peer victimization is negatively related to children and adolescents’ physical activity, that is, the higher the level of peer victimization, the lower the level of physical activity of children and adolescents.

Peer victimization may be associated with physical activity of children and adolescents through self-esteem, body self-concept or the mediating effect between self-esteem and body self-concept. That is, the higher the level of peer victimization, the lower the level of self-esteem and body self-concept, and the lower the level of physical activity.

## Data Availability

The raw data supporting the conclusions of this article will be made available by the authors, without undue reservation.
